# Noise levels in tele-operated robotic-assisted surgery—a phase-based analysis

**DOI:** 10.1007/s11548-026-03623-1

**Published:** 2026-04-18

**Authors:** Jonas Fuchtmann, Alissa jell, Maximilian Berlet, Helmut Friess, Dirk Wilhelm, Luca Wegener

**Affiliations:** 1 Research Group MITI, TUM University Hospital, Technical University of Munich, TUM School of Medicine and Health, Munich, Germany; 2 Department of Surgery, TUM University Hospital, Technical University of Munich, TUM School of Medicine and Health, Munich, Germany

**Keywords:** Noise level, Sound level, Robotic surgery, Tele-operated surgery, Ergonomics

## Abstract

**Purpose:**

Robotic-assisted surgery (RAS) is becoming increasingly popular with one of its praised benefits being improved ergonomics. While other types of interventions like open or laparoscopic surgery have been assessed regarding the noise exposure and evaluations regarding the ergonomics are based on these findings, there are no studies analyzing the changed environment of tele-operated RAS.

**Methods:**

Twelve robotic interventions with an average length of 3.5 h, including (hemi-)colectomies, anterior rectum resections and sigmoidectomies were assessed using a sound level meter. Recordings were monitored by a medical expert, manually annotating individual phases of the intervention from the patient’s entry till the exit of the operating room. Measured sound data were afterward mapped to one out of six corresponding surgical phases.

**Results:**

The data showed an average noise level of 60.5(±3.35) dB(A) and a L(A)eq of 63.49 dB(A) with the robotic phase being significantly, but only 0.15 dB(A) louder than the non-robotic surgical part (60.4(±3.14) dB(A)). Overall the peri-surgical phase was louder than the surgery itself with the post-surgical phase being the loudest of all phases with a level of 61.2 (±4.82) dB(A).

**Conclusions:**

Regarding sound levels tele-operated RAS seems comparable with open and laparoscopic surgery, despite the robotic phase being to a small extent louder within this evaluation. Nevertheless sound levels appear high and further reduction should be considered in order to improve ergonomics and patient safety.

## Introduction

Back in the year 1972 Shapiro et al. published in the New England Journal of Medicine the very first study regarding noise levels in the operating room (OR). They measured a level of 90 dB(A) during the analyzed intervention—a open cholecystectomy—and stated regarding the surgical environment "it could not be designed to be more noisy by intent" (Shapiro [1]).

Since then technology has made a great leap forward and various studies have collected data regarding sound levels and their impact. In 2007 for example, Kracht et al. measured the sound level of 38 interventions of various disciplines, including 9 not further specified visceral surgeries. The latter ones ranged thereby from 55 dB(A) up to 69 dB(A) with an L(A)eq of 63 dB(A) [2]. Further studies, also comprising data of multiple disciplines, additionally payed attention to the temporal development of the sound level during the course of the intervention. It was thereby proven, that especially the pre-surgical and post-surgical phase of the overall intervention are on average louder than the actually surgery itself [3–5]. One of the most comprehensive and recent data acquisitions analyzed 599 visceral surgeries in relation to their temporal evolvement. The results showed among other findings an average level of 61.0 dB(A) for rectum resections and 59.4 dB(A) for colon resections. Furthermore, the publication also states that laparoscopic procedures are on average 0.7 dB(A) louder compared to open procedures [6].

In recent years robotic-assisted surgery (RAS)—systems gained popularity and their availability is ever increasing. Hence, in the literature the impact of assistive systems for orthopedic interventions has already been evaluated. L(A)eq values between 88.88 dB(A) and 134.48 dB(C) have thereby been measured, depending on the used system [7]. However in comparison to orthopedic robots, to this day there is hardly any data available regarding the use of the wide spread type of tele-operated RAS systems, such as the daVinci (Intuitive Surgical Inc., Sunnyvale, United States) or Hugo RAS (Medtronic, Dublin, Ireland). Only a few selective measurements have been made, such as by Leitsmann et al. for individual timestamps during robotic prostatectomies (Skin incision: 66.54 (± 5.24) dB(A), Docking of the robot: 64,86 (± 4.09) dB(A), Incision of the bladder: 65.12 (± 4,62) dB(A), Undocking of the robot: 64.79 (± 4.39) dB(A)) [8]. Similarly Sutkin et al. measured the noise level of various individual technical devices when assessing the speech communication interference in the robotic operating room. Here levels between 64 dB(A) and 75 dB(A) have been stated when bringing a measurement probe close to devices such as the robot, console or insufflator [9].

Despite these results, the published single values for individual points in time make it impossible to draw a picture of the actual soundscape during tele-operated robotic interventions, especially in relation to non-robotic surgical phases. With the often claimed improved ergonomics of RAS and the increasing research regarding the use of audio and speech signals for automatization, it seems vital to capture baseline data of the actual sound levels [10–13]. Furthermore, previous non-RAS related studies hardly differentiated between open or laparoscopic interventions, as well as open phases in laparoscopic surgery. A high quantity of data was prioritized over the actual quality and measurement probes were placed unattended over multiple days or even weeks, making an actual surgical phase related interpretation impossible. This study therefore aims to provide better insights by capturing continuous real live sound levels of abdominal surgeries using a tele-operated RAS setup, while putting it in direct relation to the current phase of the individual intervention.

## Methods

**Fig. 1:**
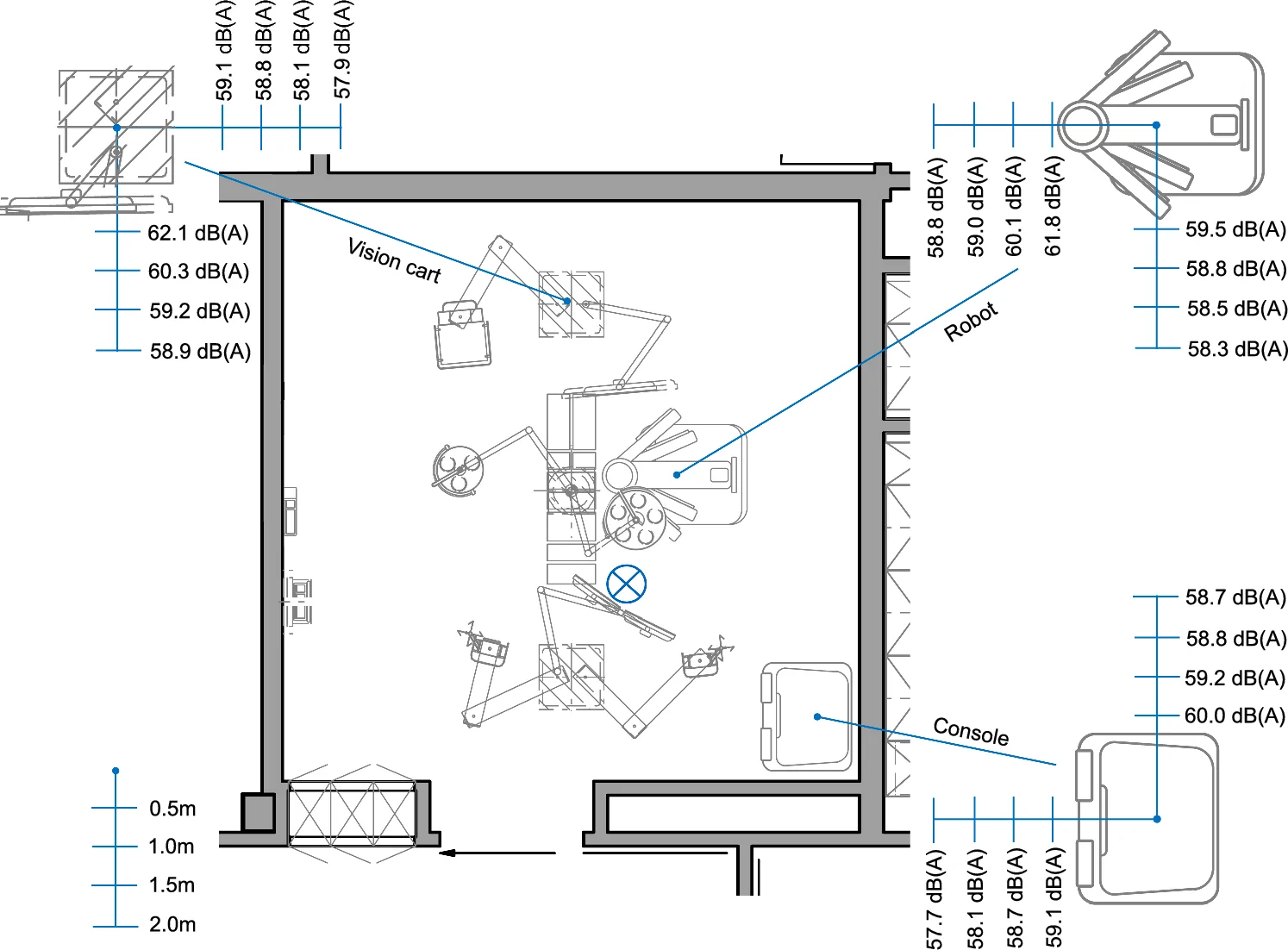
**a** Floor plan of the robotic OR of the TUM university hospital, Rechts der Isar. The position of the measurement device in relation to the rest of the OR is marked with a blue cross. Measurements for the robotic setup are given for 0.5 m, 1 m, 1.5 m and 2 m distance. Sketch not true to scale

For all measurements a calibrated class two sound level meter (PCE-322A, PCE Deutschland GmbH, Meschede, Deutschland) with an omnidirectional electret condenser microphone was used. The device itself is battery powered and can be freely positioned within the OR without interfering with the surgical workflow, due to additionally needed cables. The used version of the device is able to internally store up to 32700 individual measurements thus being able to record even long procedures. All measurements were performed in the same OR at the TUM University Hospital, Rechts der Isar (Technical University of Munich, Germany). The OR was chosen due to the daVinci Xi robot being installed in this room. Recordings took place during the day-shifts with varying staff. All parties were asked to work in their normal routine and thus measurements also included non-medical chats or for example music playing in the background. Number of attendees varied between three and eight, depending on the phase and shift. Parameters of the sound meter were set to a measurement range from 30 to 130 dB with A weighting and the device’s lowest possible measurement interval of one second. The sound meter itself was positioned on a tripod in a height of 1.5 m and in about 1 m distance to the right side of the patient’s head. This position was chosen as it sits right between the surgeon at the console, the anesthetist and the surgical assistant and thus resembles the exposure of the main OR members. Figure 1 shows the set up within the room.

**Fig. 2:**
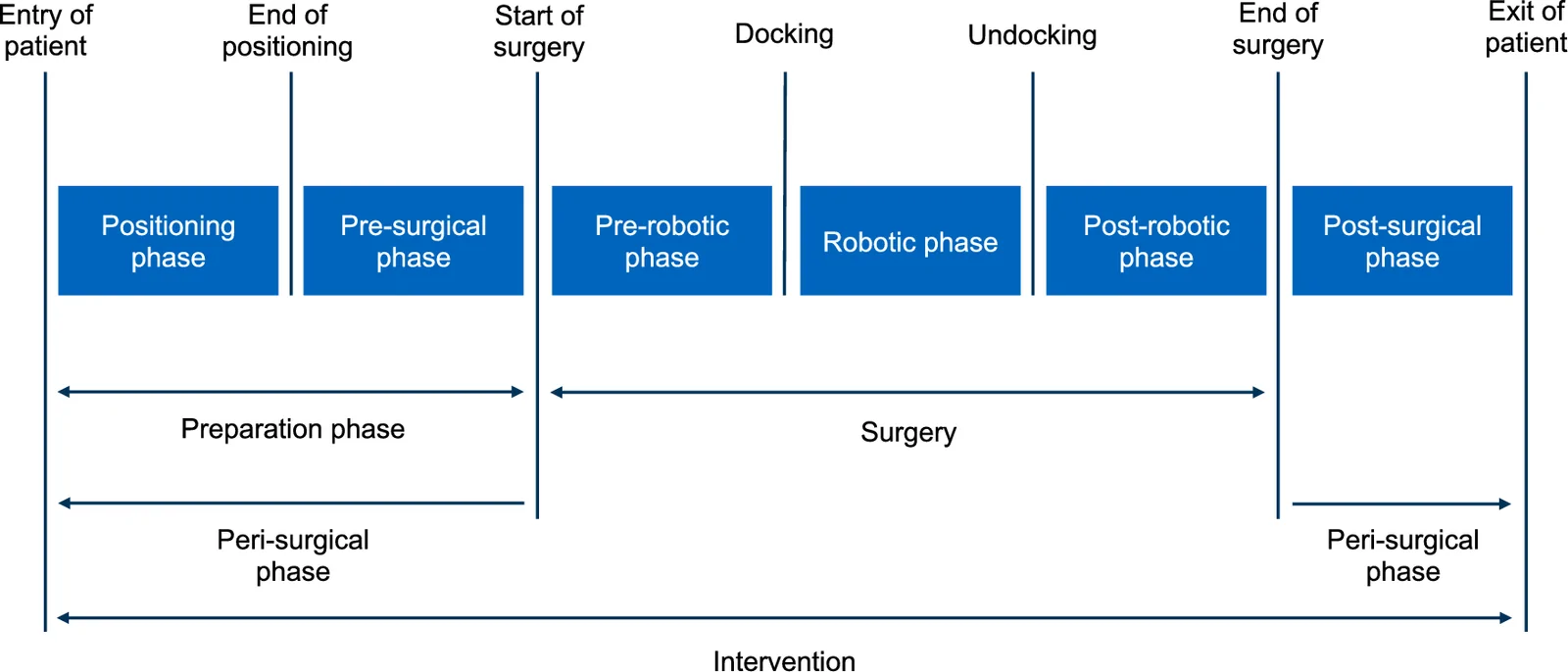
Overview of the manually annotated timestamps by the observer. Based on these multiple phases were defined and results of the measurements correlated

With 40.9 square meter the room itself has a representative size for an OR. Walls are covered with hard plastic sheets, the floor is made from polyvinyl chloride and the ceiling consists of a laminar air flow and dry wall. During each recording, an medical expert observer was manually annotating relevant timestamps in order to reference measurements to the ongoing phase of the intervention. Recordings started with the patient entering the OR. Once the patient was correctly positioned, the so-called pre-surgical phase started. Within this time period the disinfection of the skin as well as the sterile draping was executed. The actual surgery then started with the incision of the skin. The next phase was defined by the coupling of the robot to the trocars and continued until the robot was decoupled again. Once the skin was fully closed, the end of the surgery was annotated. The measurement stopped after the post-surgical phase when the patient left the OR. Figure 2 depicts the defined annotation points and the corresponding phases. In total 16 interventions were recorded, including (hemi-)colectomies, anterior rectum resections as well as sigmoidectomies. Due to device errors and missing values within the data set only 12 interventions were usable. As described in Sect. Results the ultimately analyzed data covers approximately 2513 min and contains 150804 individual measurements. An overview of the data is given in Table 1. As depicted, the individual interventions vary in their length (MEAN Duration 209 min, STD 29 min) and different points in time therefore refer to different phases. Hence, a direct one to one comparison is not meaningful. As a consequence data of each phase for each intervention was normalized with the start of a phase corresponding to the 0% mark and the end of a phase corresponding to the 100% mark. Temporal progressions for whole interventions were then stitched together using the individually normalized phases.

Additionally, as depicted in Fig. 1, individual measurements of the robotic system were taken in an empty OR. For this purpose the system was switched on, but was not actively moving. Measurements were taken from the center of each device in a distance to the level meter of 0.5 m, 1.0 m, 1.5 m and 2 m.

The statistical analysis was performed using the software R (version 4.5.2) [14] with nonparametric testing, based on the initial results of Sect. Results.

**Table 1:** Overview of the captured data per intervention, aggregated for each intervention typ and aggregated for all interventions together

Recording No.	Intervention	# Measurements	Minimum	Maximum	Range	Mean	STD	SE	IQR	Q1-1,5*IQR	1. Quartile	Median	3. Quartile	Q3+1,5*IQR
1	Total colectomy	13137	54.6	84.0	29.4	60.63	3.14	0.027	3.5	53.25	58.5	59.9	62.0	67.25
2	Right hemicolectomy	14820	55.4	84.0	28.6	60.56	2.80	0.023	2.7	54.65	58.7	59.7	61.4	65.45
3	Right hemicolectomy	12401	51.3	107.3	56.0	59.99	3.35	0.030	3.5	52.45	57.7	59.2	61.2	66.45
4	Sigmoidectomy	14288	55.2	92.0	36.8	59.86	3.28	0.027	3.8	51.8	57.5	58.9	61.3	67.00
5	Anterior rectum resection	9434	53.8	87.2	33.4	61.67	3.78	0.039	5.1	51.05	58.7	61.1	63.8	71.45
6	Sigmoidectomy	9013	55.0	88.0	33.0	60.36	3.33	0.035	3.5	52.85	58.1	59.5	61.6	66.85
7	Anterior rectum resection	13342	52.0	86.4	34.4	59.63	3.09	0.027	3.3	52.75	57.7	58.9	61.0	65.95
8	Right hemicolectomy	12360	53.3	82.7	29.4	60.72	2.87	0.026	2.7	55.05	59.1	60.1	61.8	65.85
9	Anterior rectum resection	13383	54.0	87.0	33.0	59.61	3.32	0.029	3.6	52.0	57.4	58.5	61.0	66.40
10	Sigmoidectomy	12924	56.2	95.0	38.8	61.23	3.28	0.029	3.4	53.9	59.0	60.4	62.4	67.50
11	Right hemicolectomy	11387	55.4	89.4	34.0	60.74	3.12	0.029	2.5	55.15	58.9	59.9	61.4	65.15
12	Left hemicolectomy	14315	56.2	82.0	25.8	61.36	3.97	0.033	5.3	50.35	58.3	60.0	63.6	71.55
	All (hemi-)colectomies	78420	51.3	107.3	56.0	60.68	3.27	0.012	3.4	53.40	58.5	59.8	61.9	67.00
	All sigmoidectomies	36225	55.0	95.0	40.0	60.47	3.35	0.018	3.8	52.40	58.1	59.7	61.9	67.60
	All anterior rectum resections	36159	52.0	87.2	35.2	60.16	3.48	0.018	4.1	51.55	57.7	59.2	61.8	67.95
	All interventions	150804	51.3	107.3	56.0	60.50	3.35	0.009	3.6	53.35	58.3	59.7	61.6	66.55

**Fig. 3:**
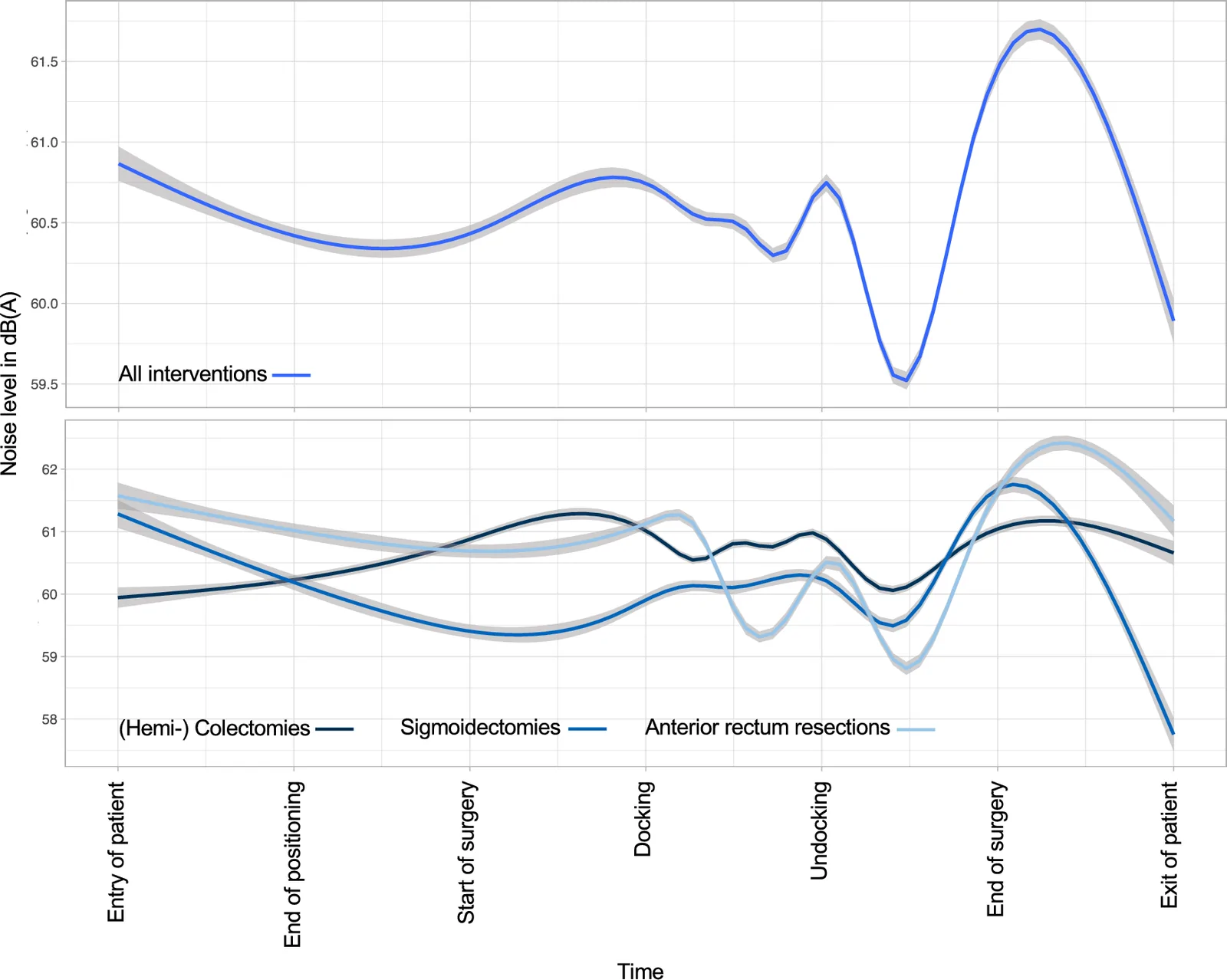
Visualization of the sound level progression throughout the interventions. Upper level graph showing an aggregated version for all the captured data, lower level graph showing a split per intervention type. 95% confidence intervals are depicted in gray. Durations of individual phases were normalized per intervention before staking phases for the full graph

## Results

The results depicted in Figure 1 show a rising level of noise with increasing proximity to the robotic devices. Especially in 50 cm distance to the front of the vision cart values of 62.1 dB(A) and above were measured, making it the loudest part of the setup. Within the captured data of the interventions, the lowest recorded level was at 51.30 dB(A) during the pre-robotic phase of intervention three, a right hemicolectomy. Within the same intervention but in the post-robotic phase 107.30 dB(A) marked the highest overall noise level of the entire data set due to the lid of a sterilization container dropping to the floor. The overall average is 60.5(± 3.35) dB(A), with the median being at 59.7 dB(A) and thus lower than the average. The levels result in an overall L(A)eq of 63.49 dB(A). The right-skewed distribution was proven graphically and by an Anderson Darling Test with *p*<0.01 for α=0.05. This right-skewed distribution also applies to each intervention individually based on quantitative tests and graphical visualizations. The average sound level for the sigmoidectomies is at 60.47(±3.35) dB(A), for (hemi-)colectomies at 60.68(±3,27) dB(A) and for anterior rectum resections at 60.15(±3.48) dB(A). Based on a Wilcoxon Rank Sum Test the overall differences between the individual types of interventions can be considered significant (*p*<0.001, α=0.05). Hence the captured (hemi-)colectomies are significant louder than the sigmoidectomies and those themselves are louder than the anterior rectum resections.

**Fig. 4:**
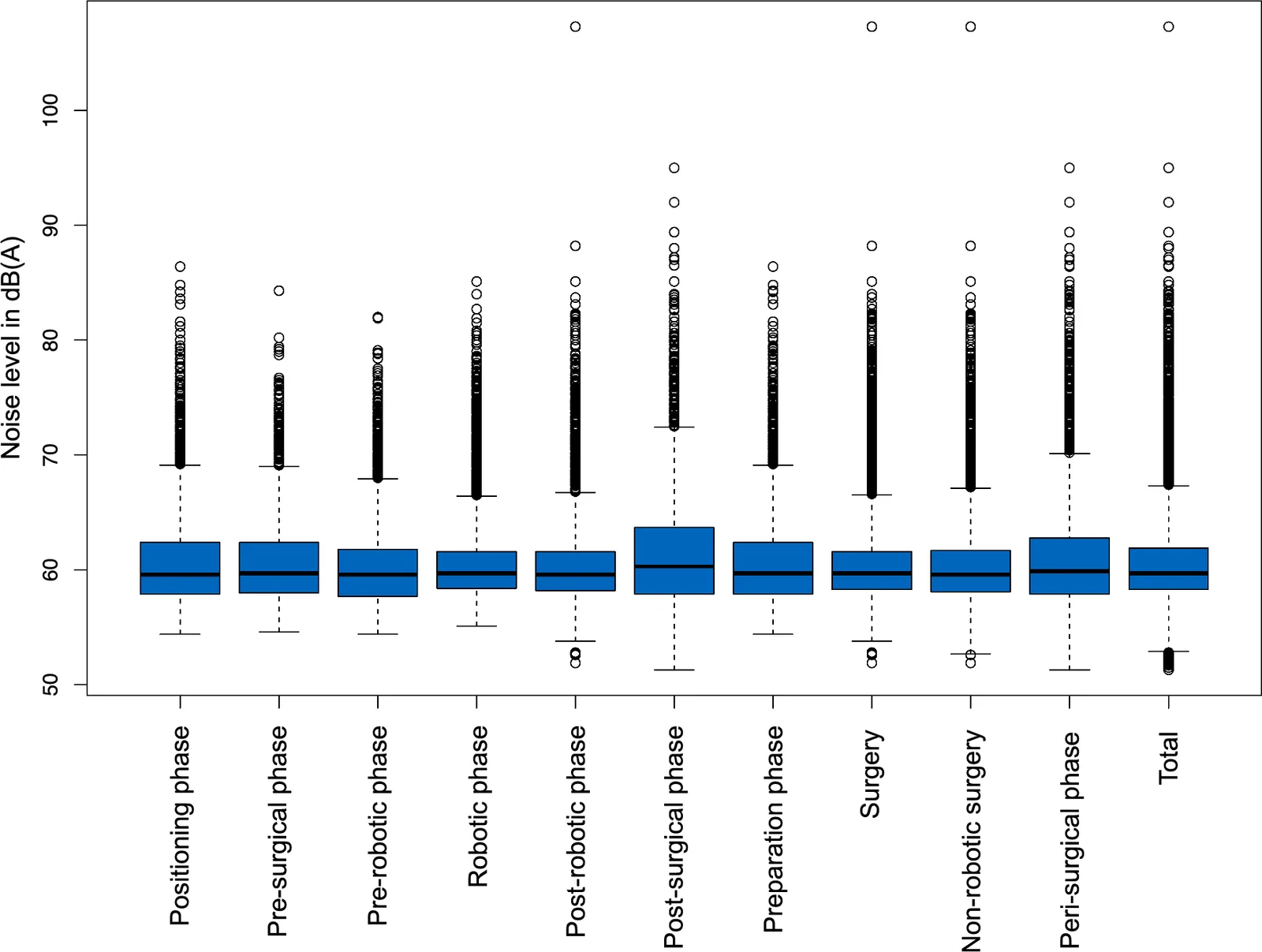
Boxplot of the measured noise levels, aggregated for all interventions and split for the individual phases. Blue boxes show the interquartile range (IQR) with the median in between. Whiskers are based on 1.5 times the IQR value and the circles depict individual data points that exceed this range

**Table 2:** Overview of the captured data per phase, aggregated for each intervention type and aggregated for all interventions together

Average(±STD) [dB(A)]	(Hemi-)Colectomies	Sigmoidectomies	Rectum resections	All interventions
Positioning phase	60.1 (±3.47)	61.3 (±3.83)	60.6 (±4.17)	60.6 (±3.35)
Pre-surgical phase	60.6 (±3.35)	60.8 (±3.68)	60.0 (±3.49)	60.5 (±3.83)
Pre-robotic phase	60.8 (±3.14)	60.6 (±3.26)	59.1 (±3.56)	60.2 (±3.39)
Robotic phase	60.7 (±3.16)	60.3 (±2.94)	60.2 (±2.94)	60.5 (±3.07)
Post-robotic phase	60.6 (±3.05)	60.0 (±3.17)	60.3 (±3.38)	60.3 (±3.17)
Post-surgical phase	61.2 (±4.65)	62.0 (±4.65)	60.6 (±5.14)	61.2 (±4.82)
Preparation phase	60.4 (±3.41)	61.1 (±3.77)	60.3 (±3.88)	60.6 (±3.64)
Surgery	60.7 (±3.13)	60.2 (±3.05)	60.1 (±3.20)	60.4 (±3.14)
Non-robotic surgery	60.6 (±2.98)	60.1 (±3.17)	60.0 (±3.46)	60.4 (±3.14)
Peri-surgical phase	60.6 (±3.83)	61.4 (±4.12)	60.4 (±4.36)	60.8 (±4.07)
Complete intervention	60.7 (±3.27)	60.5 (±3.35)	60.2 (±3.48)	60.5 (±3.35)

All results are showing the phase average and standard deviation in the unit dB(A)

**Fig. 5:**
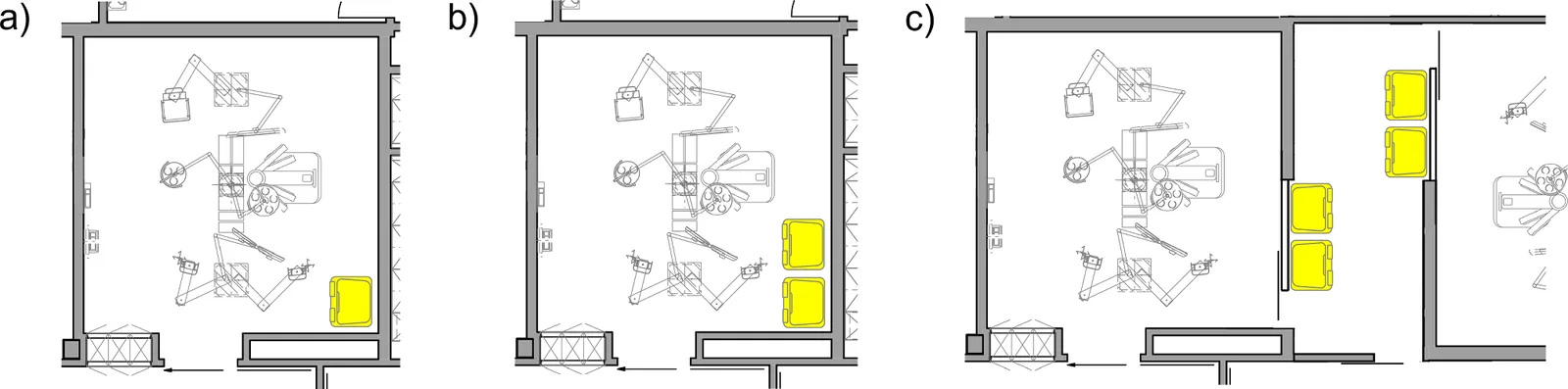
Visualization of possible adaptations for future OR configurations. **a** Shows the currently most often deployed setup with (**b**) showing the often pursued setup with an additional console for training. **c** Depicts a vision with consoles and ideally the processing unit relocated to an adjoining control room

Based on the annotations and the normalizations, total progression of the individual interventions can be compared. Figure 3 shows regression curves for the three different types of surgeries as well as for all interventions combined. The gray area thereby resembles the 95% confidence interval for the regression curves. They all show rising levels during the second half of the pre-robotic phase, fluctuate within the surgical phase and decline afterward before having again a maximum at the end of the surgical phase. Despite these common features, especially in the beginning of the recordings the ascending behavior of the (hemi-)colectomies differs from the other interventions.

For a quantitative comparison of the defined phases, data of all interventions was aggregated per phase(Table 2). Figure 4 depicts a boxplot giving a high-level overview. The data shows higher interquartile ranges for the peri-surgical phases compared to the surgical ones. As already assumable in Fig. 3 the post-surgical phase is significantly louder, compared to the rest (61.2 (±4.82) dB(A)). In contrast, the most quiet section is the pre-robotic phase with an average of 60.2 (±3.39) dB(A). When comparing the robotic phase (60.5 (±3.07) dB(A)) with the rest of the non-robotic surgical phases (60.4 (±3.14) dB(A)), there is statistically a significant difference, though it is only of 0.15 dB(A) on average. The fact of the robotic phase being louder also applies to each intervention individually. Furthermore the peri-surgical phases as well as the preparation phase are significantly louder than the surgical phase. The post-surgical phase is thereby louder as the preparation phase and simultaneously the loudest in general. This correlates with fact that in the post-surgical phase the circulators start packing the sterilization containers with instruments, as only a small set of defined tools is needed when wounds are being closed.

## Discussion

While this study provides for the first time in-depth insights into noise levels surrounding tele-operated RAS, it also has some limitations and results can not be directly extended to other ORs or hospitals. Due to the daVinci robot being the only available system in our center, findings cannot be generalized without further investigating other robotic set ups and analyzing the acoustic properties of the room itself. While one of the major goals was to generate data of the highest level of quality using manual annotations, this also limits the scale and thus the statistical power. Expanding the data acquisition over multiple robotic set ups, further types of interventions as well as increasing the overall amount of captured data would therefore be one of the future goals. This also ideally comprises a simultaneous recording of laparoscopic interventions, as current comparisons are only literature-based. Previous works stated that the influence of the position of the measurement microphone within the room can be neglected [6]. Even though we did not map all sides of the devices or recorded in an anechoic chamber, the numbers in Fig. 1 clearly proof, this changes for robotic interventions, as the setup itself is a relevant source of noise and the overall baseline level is influenced by the microphone’s position.

While the absolute differences between individual phases and intervention types might not seem huge, we would like to point out the logarithmic scale of the unit decibel, were an increase of only 3 dB equals a doubling of the noise exposure. Even so an assessment regarding occupational safety is out of the scope of this work it should be mentioned that captured noise levels drastically exceed the typical recommendations, as current WHO and European guidelines suggest levels of 55 dB(A) and lower for the surgical workplace [15, 16]. Literature also shows that noise levels, as high as within our study, impact the communication, workflow and cognitive flow of the surgical team members and thus present a threat to patient safety in robotic ORs. As levels are to a certain degree position-dependent, placement of the robotic devices within the OR should be reviewed when setting up systems. The overall architecture of the OR, the used materials and the acoustic design are crucial factors when optimizing the workplace regarding noise levels. Strategies such as the relocation of processing hardware, better damping or direct communication through headsets or in-ear monitoring should be considered for new ORs [9, 17]. Fig. 5 shows in c) a possible adaptation of the current setups a) and b) that physically separates the console and processing hardware from the OR.  In future, with more comprehensive data sets of noise levels and fine granular annotations of tasks even pattern recognition for privacy-preserving surgical data science can be evaluated.

## Conclusion

Based on our findings, the peri-surgical phase tends to be of higher noise levels compared to the actual surgery. This aligns with existing literature and might not come as a surprise, as instruments and devices are prepared and tidied up within this phase. However, with an average of 60.4 dB(A) even the sound levels of the surgical phase exceed occupational safety recommendations. The robotic phase is thereby on average louder than the non-robotic surgery, even though the difference only amounts to 0.15 dB(A). While this is the first study collecting continuous data for tele-operated RAS, further research is required to better understand the reasons for the comparatively high fluctuations within the robotic phase as seen in Fig. 3. Literature has shown that such kinds of environments promote communication interference between robotic team members and are a threat to patient safety and surgical efficiency. Hence improving future robotic setups as well as the design of ORs seems crucial.
